# Composition and Dominance of Edible and Inedible Phytoplankton Predict Responses of Baltic Sea Summer Communities to Elevated Temperature and CO_2_

**DOI:** 10.3390/microorganisms9112294

**Published:** 2021-11-04

**Authors:** Carolin Paul, Ulrich Sommer, Birte Matthiessen

**Affiliations:** Marine Ecology, GEOMAR Helmholtz-Centre for Ocean Research Kiel, 24148 Kiel, Germany; usommer@geomar.de (U.S.); bmatthiessen@geomar.de (B.M.)

**Keywords:** elevated temperature, elevated CO_2_, phytoplankton, Baltic Sea, morpho-functional traits, climate changes

## Abstract

Previous studies with Baltic Sea phytoplankton combining elevated seawater temperature with CO_2_ revealed the importance of size trait-based analyses, in particular dividing the plankton into edible (>5 and <100 µm) and inedible (<5 and >100 µm) size classes for mesozoopankton grazers. While the edible phytoplankton responded predominantly negative to warming and the inedible group stayed unaffected or increased, independent from edibility most phytoplankton groups gained from CO_2_. Because the ratio between edible and inedible taxa changes profoundly over seasons, we investigated if community responses can be predicted according to the prevailing composition of edible and inedible groups. We experimentally explored the combined effects of elevated temperatures and CO_2_ concentrations on a late-summer Baltic Sea community. Total phytoplankton significantly increased in response to elevated CO_2_ in particular in combination with temperature, driven by a significant gain of the inedible <5 µm fraction and large filamentous cyanobacteria. Large flagellates disappeared. The edible group was low as usual in summer and decreased with both factors due to enhanced copepod grazing and overall decline of small flagellates. Our results emphasize that the responses of summer communities are complex, but can be predicted by the composition and dominance of size classes and groups.

## 1. Introduction

Global climate change influences plankton communities by a number of shifting environmental factors. Two major factors that affect pelagic communities globally are increasing sea surface temperature (SST) and CO_2_ concentration. The atmospheric, and as a consequence the ocean surface CO_2_ concentration, is predicted to rise from current values of approximately 408 µatm to values of at least 700 µatm by the end of this century [1], leading, besides higher availability of CO_2_ for phototrophic organisms, to ocean acidification and lower availability of carbonate ions [2,3]. At the same time, SST has already increased on a global scale at an average rate of 0.05 °C per decade for the period 1900–2017 [1] and is predicted to increase further up to 2–5 °C on average by the end of the year 2100 [1,4,5]. More specifically, in the Baltic Sea, SST has already increased by 0.3–0.7 °C per decade and is predicted to rise up to 2–3 °C until the end of this century [6].

SST increase affects phytoplankton phenology and productivity and ultimately their abundance and species composition [7,8]. Temperature effects on phytoplankton, however, were shown to depend on region and season that vary in nutrient conditions and differ in community composition. Nutrient replete conditions prevail in temperate coastal regions such as the Baltic Sea in spring and autumn, while nutrient limited conditions characterize oligotrophic oceans and stratified shelf seas like the Baltic Sea in summer. In spring and autumn, temperate coastal phytoplankton are dominated by diatoms and dinoflagellates, the latter occur in autumn mainly. In response to increasing SST, these communities showed earlier onsets of blooms, and significant declines in bloom biomass [9,10,11]. Increasing SST directly affects copepods, leading to increased metabolic rates, and thus faster growth and an increased grazing pressure (top-down control) on the phytoplankton. As in spring, the diatoms, which are edible for copepods, dominate the total biomass. This temperature-induced increased grazing leads to a significant decrease in the phytoplankton biomass and involves a shift from diatom-dominated communities towards picoplankton and small nanophytoplankton dominance [9,12,13].

Temperate coastal phytoplankton communities in summer are less diatom-dominated and instead consist of pico/nano-plankton with cells <5 µm and flagellates 5–100 µm in diameter, large flagellates >100 µm in their longest axis, and of filamentous cyanobacteria. Besides flagellates 5–100 µm in diameter, these groups are inaccessible for mesozooplankton grazers. Hence, coastal summer phytoplankton is considered mainly bottom-up regulated via nutrient supply and predicted to be rather directly affected by elevated SST instead of indirectly via grazing. In fact, the few experiments with natural Baltic Sea summer communities and field data from the Central Baltic Sea (1979–2011) showed an increase in phytoplankton biomass either due to temperature driven increase in picoplankton and ciliates, the latter being grazed by copepods [14], or in smaller cells and diazotrophic filamentous cyanobacteria [15], both inaccessible to copepods. Contrasting results that showed lower biomass under elevated SST in a natural nutrient-limited summer community of Kiel Fjord [16] could be explained by unexpected high copepod abundance and thus increased grazing activity as the underlying reason.

With the exception of calcifiers, phytoplankton photosynthetic and growth rates are generally suggested to increase from rising CO_2_ concentrations [17,18,19], because elevated CO_2_ in the water reduce the loss by diffusion from the cell and thus the metabolic costs of carbon concentration mechanisms (CCM) [20,21]. However, CCM efficiency differs among phytoplankton species [22,23], groups, and size classes [24,25] and remain unclear for filamentous diazotrophic cyanobacteria [26]. In particular, larger phytoplankton species with a lower affinity for nutrient and gas uptake profit from elevated CO_2_ under nutrient deplete conditions, as the efficiency to use limiting nutrients to fix carbon seems to increase under such conditions [27,28]. Consequently, increases in biomass under elevated CO_2_ appeared coupled to a change in phytoplankton composition and/or mean cell size [28]. In spring blooms, diatom dominance shifted towards larger-sized species in oceanic [29,30] and Baltic Sea studies [31]. During Baltic Sea summer, in particular the predominating pico-eukaryotes profited from elevated CO_2_ and were proposed to be one of the main winners under future elevated CO_2_ concentrations [31]). Diazotrophic filamentous cyanobacteria like *Nodularia spumigena,* common in the western and Central Baltic summer communities, seemed unaffected in growth and biomass development [16,32,33].

Both, elevated SST and the benefit from increased CO_2,_ potentially affects phytoplankton cellular carbon to nutrient ratios (C:N:P ratios), which would alter stoichiometry and thus food quality. The picture, however, is still incomplete and the underlying mechanisms are not well understood. Observations in natural plankton communities range from increasing C:N [34] and C:P ratios [11,35] over no responses [16] to even decreased C:P ratios [36] under elevated temperature. Responses to elevated CO_2,_ importantly, show no relationship between stoichiometric community responses and species composition. That is, in a community where autotrophic dinoflagellates and chlorophytes gained in dominance in response to CO_2_, C:P ratios increased, while C:N ratios decreased [37]. In communities where rising CO_2_ selected for large-sized diatoms [29,38,39] or for nanophytoplankton [34], however, either N:P or C:N ratios or both increased.

The combined effects of elevated SST and CO_2_ on phytoplankton communities remain to a large amount unclear and studies on natural communities are still scarce. Although most of these studies propose an overall change in community composition, they largely differ in effect sign and magnitude [11,16,34,40,41,42,43], and as such provide little predictive information. In particular, the effects on communities including filamentous cyanobacteria are poorly understood, although the density and incidence of cyanobacterial blooms increase worldwide [44,45] and also in the Baltic Sea [15]. The few natural community studies from this area so far revealed no effects [16]; however, cyanobacteria biomass in these studies was considerably below summer-typical values and thus the responses potentially undetectable.

As underlined by the above described responses of phytoplankton to increased SST and CO_2_, trait-based differentiations of phytoplankton communities can be a fruitful pathway to understand the often varying and unexpected responses of communities and/or groups to interactive forces of environmental changes. Following the review by Litchman et al. [46], phytoplankton cell size is considered a ‘master trait’ that not only affects nutrient uptake, light absorption, sinking, and metabolic rates, but also the interaction with grazers. Cell size for instance allows to differentiate between edible and inedible protists for mesograzers and as such to predict, whether for example, temperature effects on phytoplankton biomass development are direct or indirect via changes in top-down control. For a phytoplankter, a medium-size range from 5–10 µm up to 100 µm cell length for instance means being subjected to the strongest grazing pressure by mesozooplankton copepods [47,48,49]. Communities dominated by these size classes are thus supposed to decline in biomass due to the indirect temperature effect. Knowledge about which species and size classes profit from increased CO_2_ allows to make predictions for altered trophic interrelations and thus for potential interactive effects of increased SST and CO_2_. A shift towards very small or alternatively towards larger cells can mean profound changes of the community in terms of edibility for mesograzers and bears the potential for CO_2_ and temperature effects to interact in a predictable way.

We here set out to test the combined effects of elevated SST and CO_2_ on a semi-natural Baltic Sea plankton summer community under nutrient limited to depleted conditions. We hypothesized that: (1) elevated SST in summer leads to increased total phytoplankton carbon driven by increased biomass of the dominant inedible filamentous cyanobacteria and phytoplankton <5 µm; (2) elevated CO_2_ concentration promotes growth of most phytoplankton groups and thus also increases total phytoplankton carbon; (3) the inedible phytoplankton carbon positively responds to both elevated CO_2_ and temperature; (4) the edible fraction is affected in an antagonistic manner, i.e., elevated SST declines edible phytoplankton due to enhanced grazing and CO_2_ promotes growth, and (5) both elevated CO_2_ and temperature predominantly increase elemental carbon to nutrient ratios (C:N:P ratios).

## 2. Materials and Methods

### 2.1. Experimental Design

Six CO_2_ target levels, ranging from 500 to 3000 µatm, were crossed with two different temperature regimes (13 °C, 19 °C). This resulted in twelve mesocosms, each with a volume of 1.5 m^3^ installed in four temperature-controlled culture rooms. The mesocosms contained the natural Baltic Sea late summer plankton community from Kiel Fjord (54°20’ N and 10°8’ E; 1 September 2014–26 September 2014). To realize a natural composition and density of the plankton and minimize differences in the starting community between treatments, the water was pumped from approximately two meters depth over a distributor to all mesocosms at the same time [16]. The natural community included phytoplankton (photosynthetic bacteria and algae), bacteria, and protozoa. Initial phytoplankton was dominated by large flagellates >100 µm (83%), followed by phytoplankton <5 µm (10%). As filamentous diazotrophic cyanobacteria, like *Nodularia spumigena*, mainly float directly on the water surface, they are under-represented in the depth of 1–2 m. Thus, *N. spumigena* was added as a culture in seasonal-typical concentrations to each mesocosm prior to the first sampling (culture conditions: 18 °C, temperature-controlled room, on average 150 µmol Phot L^−1^; final concentration of approximately 3.7450 cells L^−1^ per mesocosm on 1 September 2014). *Acartia tonsa* copepod nauplii (N1 stage) were added from a permanent culture to all mesocosms in a concentration of 40 ind. L^−1^, accounting for an expected mortality of 50% after addition to the mesocosms (following inoculum densities as described in Garzke et al.) [50,51]. Hatched individuals were acclimated to target temperatures, counted, and added to the mesocosms on experimental day 0 (2 September 2014; for more details see Garzke et al. [52]).

After filling, all treatments had the same temperature and CO_2_ level, consistent with the ones in Kiel Bight (16 °C, in mean 981 µatm). Over the following four days (day −4 to −1), temperature and CO_2_ were manipulated stepwise until reaching target values (day 0, fully manipulated treatments). Mesocosms consisted of swimming plastic bags (LDPE, Poly Pack), each with a surface area of approximately 1.3 m^2^ and containing approximately 200 L of Baltic Sea water. Each bag was swimming in a 1400 L barrel with a stirrer, containing also Baltic Sea water of the filling day. The mesocosms were covered by a PVC cover (polyvinylchloride, light permeable). The cover contained a sampling port, which remained closed between sampling events. In order to reduce phytoplankton sedimentation and to assure its homogeneous distribution over the course of experiment, water was mixed once a day before sample taking by moving a Secchi disk carefully up and down.

The temperature regimes, i.e., 13 °C and 19 °C, represented 3 °C above and below the actual water temperature of Kiel Bight (western Baltic Sea) on the filling day. They will be hereafter referred to as warm (19 °C) and cold (13 °C) regimes. The temperature treatments lie within the natural average SST and their fluctuations of the coastal western Baltic Sea in August/September, measured from 1957 to 2013 (mean temperature at 1 m depth, Boknis Eck: August: 17.75 °C (SD: 2.4); September: 15.55 °C (SD: 1.8), [53]).

We chose the following target CO_2_ levels for manipulation: 500, 1000, 1500, 2000, 2500, and 3000 µatm. The lowest CO_2_ regime (Figure S1) represented CO_2_ concentrations close to the minimum of the surface water in Kiel Bight. The higher regimes (Figure S1) represented present day maximum values in Kiel Bight (>2300 µatm, [54]). Such values are temporally reached during upwelling events in summer, when water masses are enriched with high dissolved inorganic carbon. These upwelling events are caused by strong winds from south-west, whereas, otherwise, the coastal water is seasonally stratified (high temperature and salinity gradients; [54]). CO_2_ values in between (Figure S1) conformed to predictions for coastal upwelling areas with highly temporal variable CO_2_ values [55], which even exceed the worst-case scenario forecast for open ocean surface waters [4].

After each sampling event (Monday, Wednesday, and Friday), CO_2_-enriched water (Kiel Bight, 0.2 µm filtered, stored in cool and in dark conditions, for enrichment CO_2_ saturated by bubbling with CO_2_ gas) was added to the mesocosms (using a flexible tube) to manipulate the target CO_2_ values and for balancing the natural CO_2_ drawdown due to phytoplankton primary production. The required volumes were calculated on the basis of total alkalinity (TA) and dissolved inorganic carbon (DIC) using the program CO2SYS [56].

Above each mesocosm, a computer-controlled light unit (GHL Groß Hard- und Softwarelösungen, Kaiserslautern, Germany) was installed. Each of these units consisted of 5 HIBay-LED spotlights (purpose build item of Econlux, 100 W each, see also Paul et al. [16]). Using the astronomic model of Brock [57], day length and light intensity were calculated and afterwards adjusted to the natural seasonal light patterns. Light conformed to 40% of the solar irradiance of an approximated cloudless day in this area [16]. The light:dark cycle was 13 h:40 min:10 h:20 min with a simulated sundown and sunrise of approximately 3.5 h. Mean maximum light intensity was 391.5 µmol photons m^−2^ s^−1^ at the water surface and 275.15 µmol photons m^−2^ s^−1^ in the middle of the water column (0.34 m below surface; LICOR Li-250A light meter, LI-COR GmbH Bad Homburg, Germany; measured 18 September 2014).

### 2.2. Sampling and Measurements

Water temperature and salinity were measured daily. Samples for DIC, TA, phytoplankton species composition and carbon biomass (including flow cytometer and microscope counting), dissolved inorganic nutrients (total dissolved inorganic nitrogen (total dissolved N), phosphate (PO_4_^3−^), silicate (SiO_4_^−^)), and particulate organic carbon (POC) were taken three times a week (Monday, Wednesday, and Friday). Experiments were finished after 24 days.

Carbonate system: DIC samples were gently pressure-filtered (0.2 µm, Sarstedt Filtropur) and collected into 50 mL gas tight vessels with at least 100 mL of overflow before sample collection. Samples were measured following the protocol of [58] with a SRI-8610C 3 (Torrence, CA, USA) gas chromatograph. For TA, 25 mL samples were filtered (Whatman GF/F filter 0.2 μm) and titrated at 20 °C with 0.05 M HCl-solution [59,60] in an automated titration device (Metrohm Swiss 6 mode; Herisau, Switzerland). To correct for any drift during analyses within a run, we used certified reference material provided by Andrew Dickson (Scripps Institute for Oceanography of the University of California, San Diego, CA, USA). The remaining carbonate parameter CO_2_ was calculated using the program CO2SYS [56,61]. Here, the constants supplied by Hansson [62] and Mehrbach et al. [63] that were refitted by Dickson and Millero [64] and the KSO_4_ dissociation constant from Dickson [65] were used for calculation. The calculated CO_2_ values are given in Figure S1.

Phytoplankton composition and carbon biomass: For the abundance of phytoplankton <5 µm, three mL of pre-filtered water (64 µm mesh) were fixed with formalin in a cryovial, flash frozen in liquid nitrogen, and kept frozen at −80 °C until measurement on a flow cytometer (FASCalibur, Becton Dickinson; Becton, Dickinson and Company, Franklin Lakes, NJ, USA). Samples were measured between 1.5 and 5 min, depending on phytoplankton <5 µm density. The discriminator was set for pigment auto-fluorescence, and bivariate plots of FSC and auto-fluorescence were used to distinguish the different populations. To determine larger phytoplankton abundance, i.e., species >5 µm, 100 mL of sample was fixed with Lugol‘s iodine and stored in the dark. Species were counted and identified at species level under an inverted light microscope, using the Utermöhl technique [66].

Total phytoplankton carbon (total phytoplankton C) calculation: The biovolume of each species (identified by flow cytometry and microscopy) was calculated by taking the respective nearest geometric standard [67]. Species’ biovolumes were converted into carbon content following Menden-Deuer and Lessard [68], i.e., C = 0.288V^0,811^ for diatoms and C = 0.216V^0.939^ for other phytoplankton (C = carbon content in pg, V = cell volume in µm^3^). Due to the fact that 180 µm^3^ is the smallest cell size included in the analysis of Menden-Deuer and Lessard [68], non-linear models would predict unrealistically high C content for smaller algae. Therefore, we followed Sommer et al. [69] and used cells below 180 µm^3^: conversion factors 0.108 pg C µm^−^^3^ for diatoms and 0.157 pg C µm^−^^3^ for all other organisms. At the end, the calculated carbon content for each species/phytoplankton group was multiplied with its respective cell abundance.

Mesozooplankton abundance: Copepods were sampled at the last day, using a hand-held plankton net (64 µm mesh size), fixed with Lugol’s iodine and later counted and identified to the developmental stages (nauplii and copepodite stage 1 to adult) and sexes (male/female, for more detail see Garzke et al. [52]).

Dissolved inorganic nutrients: For total dissolved N (including ammonium (NH_4_^+^) and nitrite/nitrate (NO_2_^−^/NO_3_^−^)), PO_4_^3−^, and SIO_4_^−^ concentrations, 20 mL sample water was filtered through cellulose acetate filters (Sartorius, 0.2 µm pore size) and immediately frozen at −20 °C. Samples were measured with an auto-analyzer (Skalar, SAN^PLUS^; Breda, The Netherlands), following the protocols of Hansen and Koroleff [70]. The detection limit of the auto-analyzer is defined as a concentration of 0.1 µmol L^−1^.

Particulate organic matter: For POC, particulate organic nitrogen (PON) and particulate organic phosphorus (POP) in total 100–250 mL water (volume depended on plankton density) were filtered onto pre-washed (in 5–10% HCl) and pre-combusted (6 h, 550 °C) Whatman GF/F filters and immediately frozen after sampling at −20 °C. POC and PON were simultaneously measured by an element analyzer (Thermo Scientific Flash 2000, Therma Fisher Scientific, Waltham, MA, USA). POP was measured colorimetrically at 882 nm [70]. Out of these measurements, molar ratios (mol:mol) were built up among POC:PON (C:N), POC:POP (C:P), and PON:POP (N:P).

### 2.3. Data Analysis

**Figure 1:**
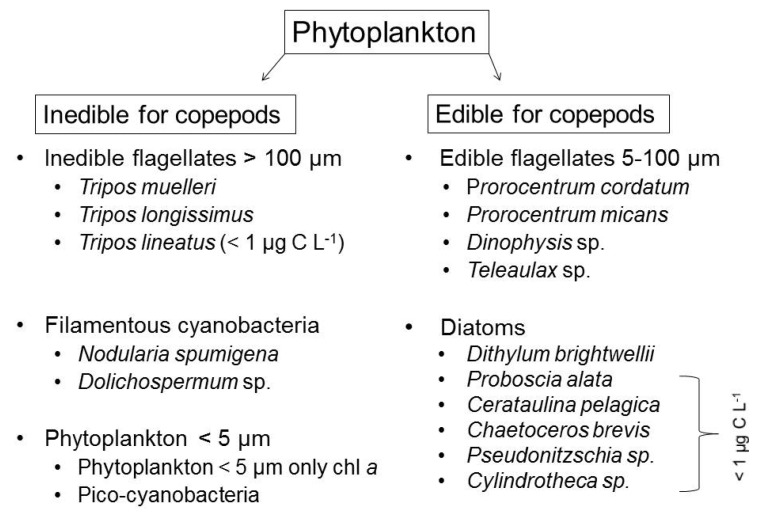
Overview of phytoplankton species composition. To account for feeding relationships between phytoplankton and mesozooplankton copepods, the phytoplankton was divided into two groups: inedible for copepods and edible for copepods. Species with a mean biomass <1 µg C L^−1^ were in the following not separately analyzed for species-specific treatment affects.

To account for indirect temperature effects via feeding relationship, total phytoplankton C and species composition were separated into two groups according to edibility for mesozooplankton (mainly copepods): inedible and edible phytoplankton (see Figure 1). The inedible phytoplankton included taxa that are known to be less preferred by copepods [47,49,71], i.e., inedible flagellates >100 µm longest axis, filamentous cyanobacteria, and phytoplankton <5 µm. In the following carbon of the inedible group will be referred to: inedible phytoplankton carbon (inedible phytoplankton C). The edible part of the phytoplankton included groups and belonging species, which are valid as edible for copepods, i.e., edible flagellates 5–100 µm and diatoms. Carbon of the edible group will be referred in the following to: edible phytoplankton carbon (edible phytoplankton C). Both edible and inedible species with a very low mean biomass <1 µg C L^−1^ were excluded from species-specific analyzes in the Supplementary Materials. As in some but not all treatments, a bloom was built-up and we divided the experimental runtime into two periods. We hereafter refer to the ‘first period’ from experimental day 3 to 13 for where in some treatments a bloom could be observed. We refer to ‘second period’ from day 14 to the end of experiment (day 24), which in the treatments with a bloom means a post-bloom phase.

### 2.4. Statistical Analyses

**Figure 2:**
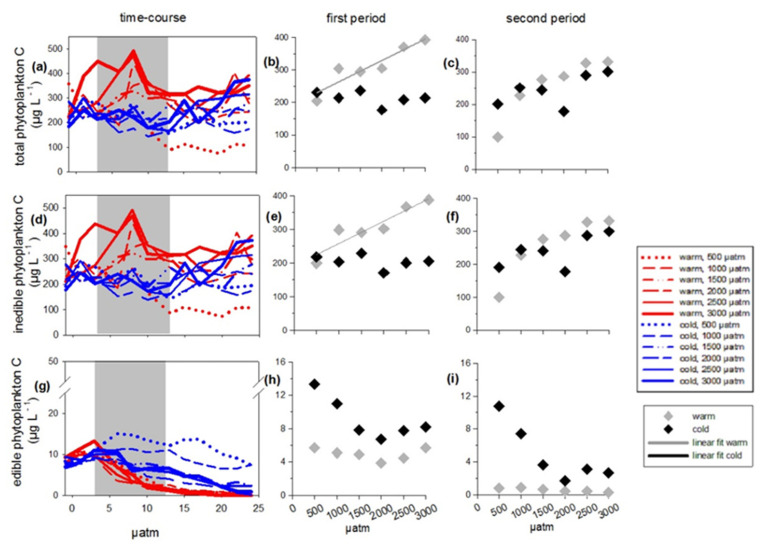
Phytoplankton carbon (µg C L ^−1^) over the entire course of time and as mean values of the first and the second period. The first period, during which a bloom in the warm treatments occurred, is marked in grey color in the subpanels (**a**), (**d**), and (**g**). (**a**–**c**) Total phytoplankton carbon, (**d**–**f**) inedible phytoplankton carbon, (**g**–**i**) edible phytoplankton carbon. Symbol attribution to treatment combinations (temperature treats + CO_2_ target values) are given in the legend. Fitted lines indicate a significant response of phytoplankton relative contribution to CO_2_ at the different temperature levels.

**Figure 3:**
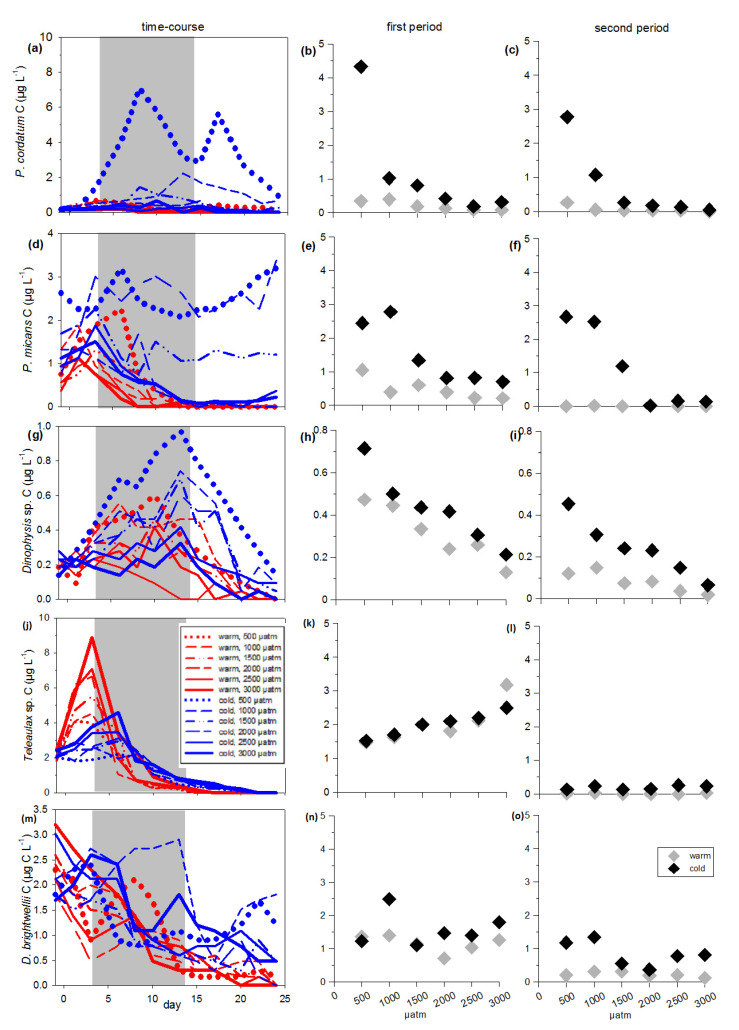
Carbon (µg C L^−1^) of the inedible and edible phytoplankton groups over the entire course of time and as mean values of the first and the second period. The first period, during which a bloom in the warm treatments occurred, is marked in grey color in the subpanels (**a**,**d**,**g**,**j**,**m**). Inedible phytoplankton groups: (**a**–**c**) flagellates >100 µm C, (**d**–**f**) filamentous cyanobacteria C, (**g**–**i**) phytoplankton <5 µm C. Edible phytoplankton groups: (**j**–**l**) flagellates 5–100 µm C, (**m**–**o**) diatom C. Symbol attribution to treatment combinations (temperature treatments + CO_2_ target values) are given in the legend. Fitted lines indicate a significant response of phytoplankton relative contribution to CO_2_ at the different temperature levels.

**Figure 4:**
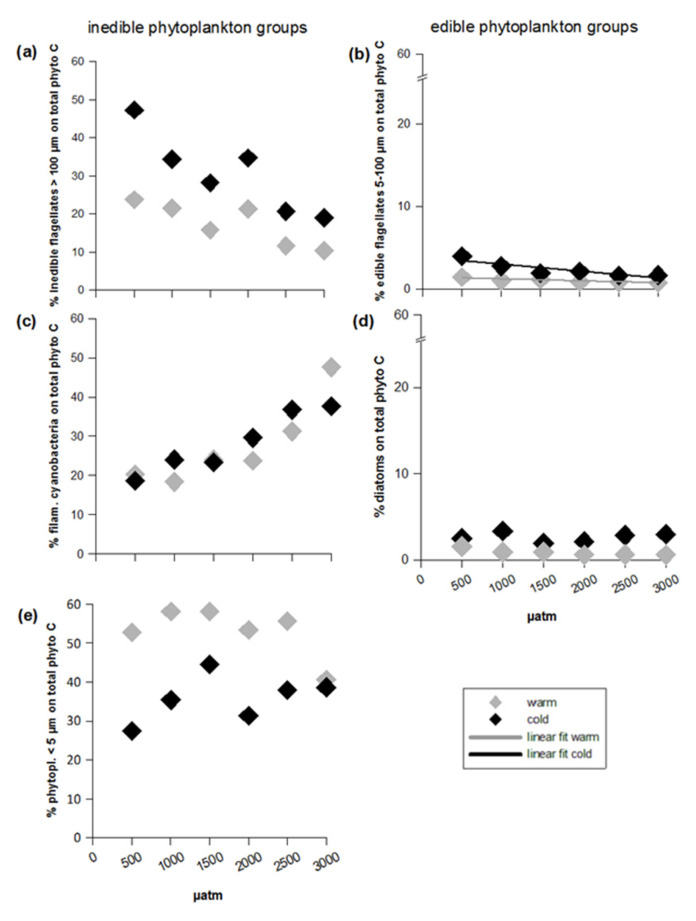
Phytoplankton relative contributions to total carbon during the first period, divided into inedible (left side) and edible groups (right side): (**a**) % inedible flagellates >100 µm on total phytoplankton C, (**b**) edible flagellates 5–100 µm on total phytoplankton C, (**c**) % filamentous cyanobacteria on total phytoplankton C, (**d**) % diatoms on total phytoplankton C, (**e**) % phytoplankton <5 µm on total phytoplankton C. Symbol attribution to treatment combinations (temperature treatments + CO_2_ target values) are given in the legend. Fitted lines indicate a significant response of phytoplankton relative contribution to CO_2_ at the different temperature levels.

To test for treatment effects during phytoplankton bloom on the measured and calculated response variables, a generalized least squares (gls) model (nlme package, R) with the factors temperature (categorical), CO_2_ (continuous) and the interactions CO_2_ x temperature was operated. As response variables, we chose: % contribution of phytoplankton groups on total phytoplankton C, total phytoplankton C, edible phytoplankton C, inedible flagellates >100 µm C, filamentous cyanobacteria C, phytoplankton <5 µm C, edible flagellates 5–100 µm C, diatom C, species-specific C biomasses, dissolved inorganic nutrient concentrations and elemental ratios (C:N:P). Statistical significant results can be found in Table A1. If a significant interaction effect was detected, we conducted a separated regression analysis with CO_2_ as continuous factor for warm and cold treatments (see Table A2, Table S4). The significant responses to CO_2_ at the different temperature levels are additionally marked in Figure 2, Figure 3 and Figure 4, Figures S2 and S3 by a regression line (linear fit warm/cold). Heterogeneity of variances was tested using Fligner-test. To test for normal distribution, all model residuals were checked using Shapiro-Wilk test and transformed (sqrt, log) if required. To take care of error distributions, contributions of phytoplankton groups on total phytoplankton C (% inedible flagellates >100 µm, % filamentous cyanobacteria, % phytoplankton <5 µm, % edible flagellates 5–100 µm, % diatoms) were traditional transformed with arcsine before statistical analyses. The level of significance (alpha) was set to 0.05. All statistical analyses were conducted using R version Ri386 3.1.0 (R Development Core Team, R Foundation for Statistical Computing, Vienna, Austria). An overview of statistical results of all factors in the models can be found in the Supplementary Tables S1–S3.

## 3. Results

### 3.1. Total Phytoplankton Carbon

The inedible fraction (Figure 1) of the phytoplankton largely dominated total phytoplankton carbon over the whole experimental time (compare Figure 2a,d). The edible fraction (Figure 1) was low in abundance as usual in summer (Figure 2g). During the first experimental period, the inedible fraction even contributed more than 90% to total phytoplankton C (Figure 4a,c,e). Both total and inedible phytoplankton carbon showed time-dependent responses to temperature and CO_2_ that were reflected in CO_2_-dependent bloom formations only in the warm temperature treatments in the first period (Figure 2a,b,d,e; Table A1, Table S1 (Supplementary Materials)). More specifically, during the first period, only in the warm treatments both total and inedible phytoplankton carbon increased significantly with CO_2_, leading to overall higher biomass in the warm treatments compared to the cold ones (Figure 2b,e; Table A1 and Table A2, Table S2 (Supplementary Materials)). During the second period, no treatment effects could be detected on total and inedible phytoplankton carbon (Figure 2c,f; Table S3). The edible fraction of the plankton did increase in biomass towards a bloom in any of the treatments and further declined in carbon content over time at higher temperature and with increasing CO_2_ concentrations, resulting in significantly lower edible phytoplankton C at elevated temperature (Figure 2g–i; Table A1, Tables S1–S3).

### 3.2. Inedible Phytoplankton Groups

The different groups of the inedible fraction showed different responses to the treatments (Figure 3a–i and Figure 4a,c,e). At the beginning of the experiment, the inedible phytoplankton predominantly consisted of large flagellates >100 µm (Figure 3a) and filamentous diazotrophic cyanobacteria (Figure 3d). However, from the first (bloom) period and over the course of the second, the dominance shifted towards phytoplankton <5 µm, still followed by filamentous cyanobacteria (Figure 3d,g). Both phytoplankton <5 µm and filamentous cyanobacteria gained from both elevated temperature and CO_2_, but in different ways. The increase in phytoplankton <5 µm was most pronounced in the warm treatments during the first period (Figure 3g,h; Table A1, Tables S1–S3), resulting in a 17% higher mean contribution to total phytoplankton carbon in the warm temperature treatments compared to the cold ones (Figure 4e). Filamentous cyanobacteria predominantly profited from elevated CO_2_ (Figure 3d–f; Table A1, Tables S1–S3). During the first period, their positive response to CO_2_ was stronger in the warm compared to the cold treatments (Figure 3e; Table A1 and Table A2), resulting in a filamentous cyanobacteria contribution of 50% to total phytoplankton carbon in the highest CO_2_ regime during bloom (Figure 4c; Table A1). Contributions of inedible flagellates >100 µm in turn significantly declined with both increasing temperature and CO_2_ (Figure 4a, Table A1, Table S2 (Supplementary Materials)). The negative response to the latter was more pronounced in the cold temperature treatments during the first period (Figure 3b and Figure 4a; Table A1 and Table A2) and persisted over the second one. As a result, large flagellates >100 µm went almost extinct in all warm temperature treatments during post-bloom in the second period (Figure 3c; Table S3 (Supplementary Materials)).

For some relevant inedible species, descriptions of specific responses to treatments can be found in the Supplementary Materials (Supplementary Figure S2; Tables S1–S4).

### 3.3. Edible Phytoplankton Groups

Just at the beginning of the first period, the edible flagellates 5–100 µm peaked in all warm treatments, but steeply declined thereafter over the ongoing bloom, resulting in carbon values close to zero in the second (post-bloom) period (Figure 3j–l). In the cold treatments, a delayed peak was found in the lowest CO_2_ concentrations (Figure 3j–l); however, flagellates responded significantly negative to increasing CO_2_ (Figure 3j–l and Figure 4b; Table A1, Tables S1–S3 (Supplementary Materials)). Diatom carbon declined mainly in response to elevated temperature (Figure 3m–o; Table A1 and Table A2, Tables S1–S3 (Supplementary Materials)). This temperature sensitivity resulted in values close to zero in all warm treatments during the second period (Figure 3o, Table S3 (Supplementary Materials)), while they showed some fluctuations in the cold treatments maintaining significantly higher carbon than in the warm ones (Figure 3m–o and Figure 4d; Table A1, Tables S1–S3).

For some relevant edible species, descriptions of specific responses to treatments can be found in the Supplementary Materials (Supplement Figure S3; Tables S1–S4 (Supplementary Materials)).

### 3.4. Dissolved Inorganic Nutrients

Total available dissolved inorganic N concentrations (nitrite, nitrate, and ammonium) were low in all treatments from the middle of the first period (1–1.5 µmol L^−1^; Figure S4a; Table S1 (Supplementary Materials)). Dissolved inorganic phosphate (PO_4_^3−^) steadily declined over time close to detection limit (0.2 µmol L^−1^) without treatment effects (besides an unexplained anomaly in warm treatments with low CO_2_ during the first period, Figure S4b; Table S1 (Supplementary Materials)). This resulted in dissolved N: dissolved P ratios (DIN:DIP) of 5 to 7.5 where PO_4_^3^^−^ was only 0.2 µmol L^−1^ and to even lower DIN:DIP ratios where P was higher (Figure S4a,b (Supplementary Materials)), indicating N-limitation. Dissolved inorganic silicate (SiO_4_^−^) decreased over the course of time but with no significant responses to treatments (Figure S4c; Table S1 (Supplementary Materials)).

### 3.5. Particulate Organic Matter Stoichiometry

The carbon to nitrogen ratios (C:N) decreased with increasing CO_2_ concentrations, (Figure S5a–c; Table A1 and Table A2, Tables S1–S3 (Supplementary Materials)), which indicates a relaxation of N-limitation under elevated CO_2_ during the first and the second period. However, ratios were still close to Redfield, indicating a low degree of limitation. Carbon to phosphorus ratios (C:P), instead, were permanently below the Redfield Ratio in all treatments (Figure S5d; Table S1 (Supplementary Materials)), indicating phosphorus-limited conditions. During the first period, C:P ratios significantly increased with increasing CO_2_ in the warm treatments (Figure S5e, Table A1 and Table A2, Table S2 (Supplementary Materials)), coinciding with the increase of filamentous cyanobacteria carbon. Nitrogen to phosphorus ratios (N:P) were below Redfield Ratio in all treatments (Figure S5g (Supplementary Materials)), reflecting nitrogen limited conditions that promote blooms of diazotrophic cyanobacteria [72,73,74]. N:P ratios increased under both temperature treatments with enhanced CO_2_ during the first period (Figure S5h; Table A1, Table S2 (Supplementary Materials)). In the second period, ratios were low (N:P < 4) in all treatments (Figure S5i; Table S3 (Supplementary Materials)).

## 4. Discussion

Phytoplankton biomass development of the Baltic Sea in summer is considered as mainly bottom-up regulated via nutrient supply and less top-down via zooplankton grazing as phytoplankton in this season mainly consists of size-classes inedible for mesozooplankton grazers [14]. Accordingly, our experimental communities were dominated by inedible phytoplankton with over 90% (Figure 4), which were responsible for the positive response of total phytoplankton carbon to elevated SST and CO_2_. Specifically, filamentous cyanobacteria and phytoplankton <5 µm increased in biomass towards a bloom in the warm treatments and thus increased total phytoplankton biomass (supporting hypothesis 1, 2, 3). The minor edible fraction did not increase in biomass towards a bloom. Moreover, in contrast to the inedible fraction, the edible phytoplankton decreased with both single factors elevated SST and CO_2_ (partly rejecting hypothesis 4). The most likely explanation is temperature-induced enhanced mesozooplankton grazing and the overall negative responses of the edible flagellates 5–100 µm to both factors. The positive response of C:P to combined elevated temperature and CO_2_ was potentially coupled with the rise in filamentous cyanobacteria (partly supporting hypothesis 5).

Our results add to the number of studies showing that the regulation of summer phytoplankton in coastal temperate regions and its consequent responses to elevated SST and CO_2_ fundamentally differs from more top-down regulated spring communities [9,12,13]. In spring it was shown that elevated temperature enhanced copepod grazing on the dominant edible fraction (i.e., diatoms), which led to a shift in species composition from diatom to a pico- and nano-sized phytoplankton and decreased total biomass [9,12,13]. Summer responses seem to be more complex and variable, likely depending on the species composition [14,15,16]. Corroborating our results, it was shown that either elevated temperature [14,15,75] or CO_2_ concentrations [31] can shift communities to a dominance of small sized species (pico-eucaryotes and pico-cyanobacteria) and large filamentous cyanobacteria and increase total phytoplankton biomass.

### 4.1. Effects of Temperature and CO_2_ on the Inedible Fraction

The observed shift in dominance within the inedible size fraction, i.e., from large flagellates >100 µm in diameter and large filamentous diazotrophic cyanobacteria to phytoplankton species <5 µm, matches previous temperature experiments with summer communities [14,75] and field data from the Central Baltic Sea (1979–2011, [15]). The widely known preference of cyanobacteria for warm waters [44] makes it probable that they will belong to one of the groups that benefit from climate warming [15]. Our results even suggest that elevated CO_2_ might strengthen this effect, as their positive responses to elevated CO_2_ during bloom were strongest in combination with elevated temperature. Both a recent meta-analysis [76] and culture studies, e.g., [77,78,79], likewise revealed positive responses of *N. spumigena* and non-heterocystous cyanobacteria in growth and C-fixation to elevated CO_2_. However, the mechanism behind this profiting, e.g., via down-regulation of costly CCM, remains to a large amount unclear for this group [26]. Natural community studies from the Baltic Sea revealed no effects on filamentous diazotrophic cyanobacteria under combined elevated temperature and CO_2_ [16] or adjusting CO_2_ as a single factor [32]. Both studies suggested that the absence of detectable CO_2_ effects was potentially based on the overall low biomass of filamentous cyanobacteria (<6% of total phytoplankton C [16]; <5 µg C L^−1^ [32]).

High CO_2_ concentrations have further the potential to increase the efficiency for larger inedible phytoplankton to use limiting nutrients to fix carbon [38,39,80]. Diazotrophic cyanobacteria are independent from dissolved inorganic nitrogen sources due to atmospheric N-fixation, but dependent on elevated P-availability due to their high P demand. Dissolved inorganic phosphorous availability was low in all treatments (PO_4_^3−^ < 0.6 µmol L^−1^); however, uptake efficiency possibly increased under elevated CO_2_. This potentially explains the coincidence of enhanced C:P ratios and high filamentous cyanobacteria C (50% to total phytoplankton carbon) in the high CO_2_ treatments (Figure 3e and Figure S5e (Supplementary Materials)). Additionally, low N:P ratios (Figure S5g–i (Supplementary Materials)) likewise may promote biomass development of diazotrophic cyanobacteria [72,73,74].

A significant fraction (35 to 80%) of the atmospherically fixed nitrogen by cyanobacteria can be directly released into the surrounding environment [81,82] and as such enhance the availability of nitrogen for other phytoplankton species. For instance, the small-sized inedible group <5 µm with their high affinity for limiting nutrients [83] may have benefitted from such additional N-source in the filamentous cyanobacterial-rich warm and high CO_2_ treatments. The small phytoplankton <5 µm dominated both total phytoplankton carbon (mean during bloom: 42%, Figure 4e) and the inedible fraction (Figure 3g–i) in the warm treatments; however the hypothesized positive responses to temperature were minor. Nevertheless, our results support the assumption of Suikkanen et al. [15] that phytoplankton communities of the Baltic Sea will proceed changing to a smaller-sized species structure in combination with filamentous cyanobacteria under ongoing increasing water temperatures in summer.

In contrast to our hypotheses, flagellates were overall negatively affected by both increasing water temperature and CO_2_ (rejecting hypotheses 1, 2, partly 3). Both edible flagellates (*Prorocentrum* spp.) and the inedible flagellate taxa *Tripos* nearly disappeared during the second (post-bloom) period in the warm treatments (Figure 3c,l; Figures S2 and S3 (Supplementary Materials)). Thus, responses seem to be both taxa/group specific and trait-based, i.e., directly treatment-induced and indirectly via grazing (in details discussed below). Our results are in line with studies from the Central Baltic Sea [84] and the Mediterranean Sea [85], where species of the taxa *Tripos* shifted occurrence from summer to late autumn or disappeared from the surface to deeper and colder water layers due to increasing SST. A 50-year time series (1960–2009) of the North Sea and the northeast Atlantic further detected a decline in dinoflagellate abundance and particularly of the dinoflagellate species *C. furca* and *Prorocentrum* spp. [86]. Following Tunin-Ley et al. [85], for instance the taxa *Tripos* sp. Responses are quite sensitive to increasing SST and are even discussed as an indicator taxa for climate change [87].

The sensitivity of non-calcifying flagellates to elevated CO_2_, instead, was to the best of our knowledge, reported only rarely. Whereas, a recent meta-analysis found an overall benefit of dinoflagellates from elevated CO_2_ [17], a literature analysis revealed a strong variation in sensitivity between taxa and species due to dinoflagellates’s diverse trophic strategies [18]. The few studies on natural communities with CO_2_ levels exceeding 1000 µatm differ in their results, ranging from negative responses under elevated CO_2_ alone and positive effects in combination with elevated SST [34] to no responses at all [16]. However, due to the low flagellate abundance in the latter one (total flagellate percentage on total phytoplankton C: 5%, not published), responses were potentially below detection limit.

### 4.2. Effects of Temperature and CO_2_ on the Edible Fraction

The edible faction of total phytoplankton C and its component groups and species (see Figure 1) were of minor importance and decreased under elevated temperature (diatoms, flagellates 5–100 µm) and CO_2_ (flagellates 5–100 µm, already discussed above). Temperature effects go in line with previous studies on natural plankton communities including mesozooplankton grazers, mainly investigating phytoplankton spring and autumn blooms, where the edible group (mainly diatoms and/or flagellates) dominates the phytoplankton community. In these studies, the decrease was explained by temperature-induced enhanced mesozooplankton copepod grazing, e.g., [12,16,88,89]. Mesozooplankton analyses of this experiment by Garzke et al. [52] detected a temperature-induced ontogenetic shift of the stage distribution of the copepod *A. tonsa*. While cold treatments were mainly adult-dominated at the end of the experiment, warm treatments showed a considerably higher number of younger stages, explained by warming-induced faster reproduction and development of a new nauplii generation [52]. Overall, this led to a significantly higher total mesozooplankton abundance in the warm treatments compared to the cold ones (warm treatments: 13–47 individuals L^−1^; cold treatments: 1–14 individuals L^−1^; *p* (temperature) = 0.0065; oral communication by Garzke, J. 2020; [52]), which considerably enhanced grazing on the edible phytoplankton fraction in the warm treatments.

## 5. Conclusions

Overall, our results reveal the importance of size-trait-based analyses of plankton communities, i.e., to distinguish between indirect responses by the edible-size group via zooplankton grazing and direct responses by the inedible-size group. We showed that complex climate-change related responses of temperate summer plankton communities can be predicted by the composition and dominance of size classes and groups. In more detail, results confirm previous suggestions that summer communities of the Baltic Sea will change to dominance of filamentous cyanobacteria and small-sized phytoplankton species under ongoing increasing SST. Particularly in high-acidified systems, filamentous cyanobacteria might increase strongly and enhance the problematic of toxic blooms. Larger flagellates, instead, potentially disappear from the Baltic summer plankton community under future climate conditions.

## Data Availability

The original data set of this research is stored at PANGAEA and will be available under: www.pangaea.de (accessed on 2 November 2021).

## Supplementary Materials

The following are available online at https://www.mdpi.com/article/10.3390/microorganisms9112294/s1, Figure S1: Time course of calculated CO_2_ values. For symbol attribution to treatment combination see legend. Figure S2: Carbon (µg C L^−1^) of inedible phytoplankton species/taxa, over the entire course of time and as mean values of the first and the second period. The first period, during which a bloom in the warm treatments occurred, is marked in grey color in the subpanels (a,d,g,k,n,q). (a–c) *T. muelleri*, (d–f) *T. longissimus*, (g–i) *N. spumigena*, (k–m) *Dolichospermum* sp., (n–p) phytoplankton <5 µm only containing chl *a*, (q–s) pico-cyanobacteria. Symbol attribution to treatment combinations (temperature treats + CO_2_ target values) are given in the legend. Fitted lines indicate a significant response of phytoplankton relative contribution to CO_2_ at the different temperature levels. Figure S3: Carbon (µg C L^−1^) of edible phytoplankton species/taxa, over the entire course of time and as mean values of the first and the second period. The first period, during which a bloom in the warm treatments occurred, is marked in grey color in the subpanels (a,d,g,k,n). (a–c) *P. cordatum*, (d–f) *P. micans*, (g–i) *Dinophysis* sp., (k–m) *Teleaulax* sp., (n–p) *D. brightwellii.* Symbol attribution to treatment combinations (temperature treats + CO_2_ target values) are given in the legend. Fitted lines indicate a significant response of phytoplankton relative contribution to CO_2_ at the different temperature levels. Figure S4: Time-course of dissolved inorganic nutrient concentrations (µmol L^−1^) of: (a) total dissolved inorganic N (NO_2_^−^/NO_3_^−^, NH_4_^+^), (b) phosphate (PO4_3_^−^) and (c) silicate (SiO_4_^−^). Symbol attribution to treatment combinations (temperature treats + CO_2_ target values) are given in the legend. The first period, during which a bloom in the warm treatments occurred, is marked in grey color. Figure S5: Plankton stoichiometry (mol:mol) over the entire course of time and as mean values of the first and the second period. The first period, during which a bloom in the warm treatments occurred, is marked in grey color. (a–c) carbon to nitrogen ratio (C:N), (d–f) carbon to phosphorus ratio (C:P), (g–i) nitrogen to phosphorus ratio (N:P). Symbol attribution to treatment combinations (temperature treats + CO_2_ target values) are given in the legend. Redfield Ratios are marked with a horizontal line in the subpanels (a,d,g). Fitted lines indicate a significant response of phytoplankton relative contribution to CO_2_ at the different temperature levels. Table S1: Results of generalized least squares model (gls) testing for the effect of temperature (T), CO_2_, time and the interaction effect of temperature and CO_2_ (T × CO_2_), time and temperature (time × T), time and CO_2_ (time × CO_2_) and the interaction of time, temperature and CO_2_ (time × T × CO_2_) over the entire course of experimental time. Significant results are in bold. * *p* ≤ 0.05, ** *p* < 0.01, *** *p* < 0.001. Table S2: Results of generalized least squares model (gls) testing for the effect of temperature (T), CO_2_, and the interaction effect of temperature and CO_2_ (T × CO_2_) during the first period. Significant results are in bold. * *p* ≤ 0.05, ** *p* < 0.01, *** *p* < 0.001. Table S3: Results of generalized least squares model (gls) testing for the effect of temperature (T), CO_2_ and the interaction effect of temperature and CO_2_ (T × CO_2_) during the second period. Significant results are in bold. * *p* ≤ 0.05, ** *p* < 0.01, *** *p* < 0.001. Table S4: Results of generalized least squares model (gls) testing for the effect of CO_2_ under high and low temperature for the edible and inedible species separately. Significant results are in bold. * *p* ≤ 0.05, ** *p* < 0.01, *** *p* < 0.001.

## Appendix A

**Table A1:** Significant results of generalized least squares model (gls) testing for the effect of temperature (T), CO_2_, time, and the interaction effect of temperature and CO_2_ (T × CO_2_), time and temperature (time × T), time and CO_2_ (time × CO_2_) and/or the interaction of time, temperature and CO_2_ (time × T × CO_2_) over the entire course of experimental time, for the first period and for the second period. * *p* ≤ 0.05, ** *p* < 0.01, *** *p* < 0.001.

Response Variable	Factor	df Residual	t-Value	*p*
Entire course of experimental time				
(log) total phytoplankton C (µg L^−1^)	time × T	136	−2.44663	0.0157 *
	time × CO_2_	136	2.27327	0.0246 *
(log) inedible phytoplankton C (µg L^−1^)	time × T	136	−2.23612	0.0270 *
	time × CO_2_	136	2.44929	0.0156 *
Edible phytoplankton C (µg L^−1^)	time × T	136	−4.37454	<0.001 ***
	time × CO_2_	136	−4.00765	<0.001 ***
Inedible flagellates > 100 µm C (µg L^−1^)	time × T	136	−2.15323	0.0331 *
(log) filamentous cyanobacteria (µg L^−1^)	T × CO_2_	136	2.231858	0.0274 *
	time × CO_2_	136	2.758938	0.0067 **
Edible flagellates 5–100 µm C (µg L^−1^)	T	136	−2.13354	0.0347 *
	CO_2_	136	−2.30590	0.0226 *
	T × CO_2_	136	2.024677	0.0449 *
	time × T	136	−2.84060	0.0052 **
	time x CO_2_	136	−2.86083	0.0049 **
Diatom C (µg L^−1^)	time × T	136	−4.12406	<0.001 ***
	time x CO_2_	136	−3.57416	<0.001 ***
	time × T × CO_2_	136	2.750142	0.0068 **
(log) C:N	CO_2_	136	−2.32444	0.0216 *
PO_4_^3−^ (µg L^−1^)	T	136	2.785997	0.0061 **
First period				
% inedible flagellates > 100 µm	T	8	−3.93871	0.0043 **
on total phytopl. C	CO_2_	8	−4.95259	0.0011 **
% edible flagellates 5–100 µm on total phytopl. C	T	8	−5.18685	<0.001 ***
	CO_2_	8	−5.02835	0.0010 **
	T × CO_2_	8	2.562598	0.0335 *
% filam. cyanobacteria on total phytopl. C	CO_2_	8	3.644613	0.0065 **
% phytopl. <5 µm on total phytopl. C	T	8	3.831281	0.0050 **
(log) inedible flagellates >100 µm C	T	8	−3.25951	0.0115 *
(µg L^−1^)	CO_2_	8	−5.12617	<0.001 **
	T × CO_2_	8	2.35489	0.0463 *
(log) filamentous cyanobacteria C (µg L^−1^)	CO_2_	8	3.320493	0.0105 *
	T × CO_2_	8	2.891799	0.0201 *
(log) C:N	CO_2_	8	3.127289	0.0141 *
C:P	T × CO_2_	8	2.586698	0.0323 *
N:P	CO_2_	8	2.491033	0.0375 *
Second period				
(log) edible phytoplankton C (µg L^−1^)	T	8	−5.00670	0.0010 **
	CO_2_	8	−3.67592	0.0063 **
(log) filamentous cyanobacteria C (µg L^−1^)	CO_2_	8	6.531437	<0.001 ****
(log) edible flagellates 5–100 µm C (µg L^−1^)	T	8	−6.69378	<0.001 ***
	CO_2_	8	−6.92440	<0.001 ***
(log) diatom C (µg L^−1^)	T	8	−3.15491	0.0135 *

**Table A2:** Results of generalized least squares model (gls) testing for the effect of CO_2_ under high and low temperature separately. Significant results are in bold. * *p* ≤ 0.05, ** *p* < 0.01, *** *p* < 0.001.

Response Variable	Factor	df Residual	t-Value	*p*
Total phytoplankton C first period (µg L^−1^)	CO_2_ warm	4	5.086691	**0.0070** **
	CO_2_ cold	4	−0.928886	0.4055
Inedible phytoplankton C first period (µg L^−1^)	CO_2_ warm	4	5.107762	**0.0069** **
	CO_2_ cold	4	−0.743826	0.4983
Inedible flagellates > 100 µm C first period (µg L^−1^)	CO_2_ warm	4	−1.571086	0.1913
	CO_2_ cold	4	5.005789	**0.0075** **
Filamentous cyanobacteria C first period (µg L^−1^)	CO_2_ warm	4	3.982850	**0.0164** *
	CO_2_ cold	4	4.209925	**0.0136** *
Edible flagellates 5–100 µm C first period (µg L^−1^)	CO_2_ warm	4	0.053824	0.9597
	CO_2_ cold	4	−3.590508	**0.0230** *
% edible flagellates 5–100 µm on	CO_2_ warm	4	−3.685677	**<0.001** ***
total phytoplankton C (µg L^−1^)	CO_2_ cold	4	−3.692101	**0.0211** *
C:N second period	CO_2_ warm	4	1.29419	0.2653
	CO_2_ cold	4	−10.16259	**<0.001** ***
C:P first period	CO_2_ warm	4	2.761417	**0.0508** *
	CO_2_ cold	4	0.042495	0.9681
