# Supplementary material for: Composition and Dominance of Edible and Inedible Phytoplankton Predict Responses of Baltic Sea Summer Communities to Elevated Temperature and CO2
Source: Microorganisms. 2021 Nov 4;9(11):2294. doi: 10.3390/microorganisms9112294 (PMC8621663; doi:10.3390/microorganisms9112294)
Supplement: Supplementary file 1 [file microorganisms-09-02294-s001.zip › microorganisms_1390211 Supplementary figures.pdf]

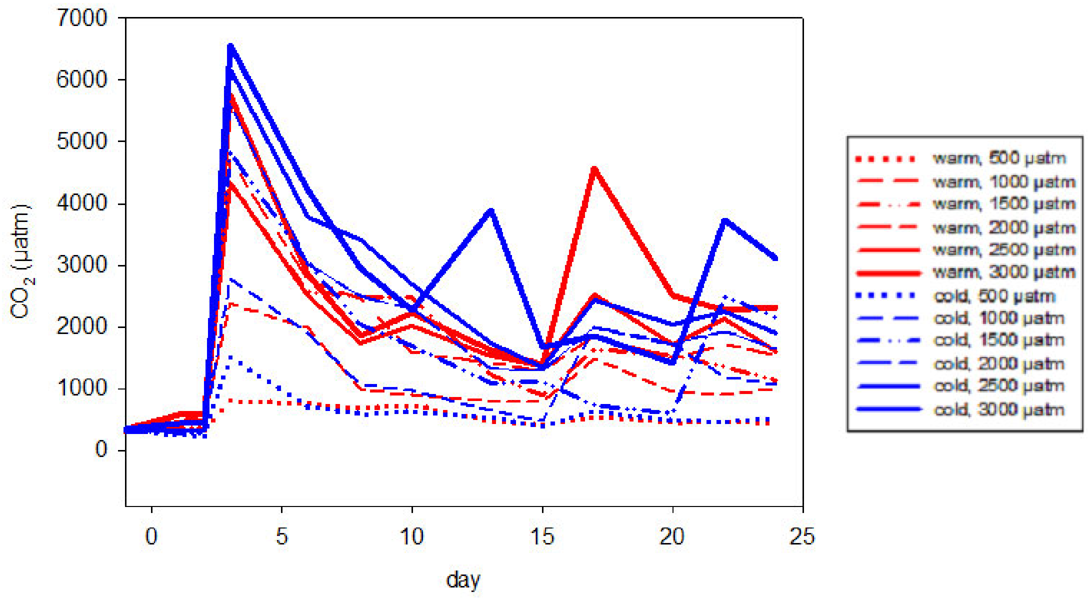

Figure S1. Time course of calculated CO_2 values. For symbol attribution to treatment combination see legend.

Supplement material*: Species / groups of the inedible phytoplankton fraction:*

The flagellates *T. muelleri* and *T. longissimus* decreased strongly over the entire course of time in all treatments (Fig. S2a, d; Table S1). Whereas species negatively responded to increasing CO_2 in the cold treatments (Fig. S2 a-f; Table S1-S3), the negative response to temperature lead to carbon biomass close to zero in the warm treatments in the second period (Fig. S2 a-f, Table S1-S3). The filamentous cyanobacteria *N. spumigena*, instead, positively responded to elevated CO_2 levels under both temperature regimes during both, the first and the second period, however with stronger effects in the warm treatments during the first period mainly triggered by the 2500 and 3000 µatm mesocosms (Fig. S2 g-i; Table S1-S4). *Dolichospermum* sp. carbon increased slightly during the first period and reached peaks in the second period, but without differences between treatments (Fig. S2 k-m; Table S1-S3). Carbon of the phytoplankton < 5 µm only containing chl *a* increased strong during the first period in the warm mesocosms (mean: 167 µg C L^{-1}; Fig. S2 o) but without any significant treatment effects (Fig. S2 n-p; Table S1-S3). Pico-cyanobacteria carbon was significantly, but only minor, higher in the warm treatments compared to the cold ones during the first period but not during the second period (Fig. S2 q-s; Table S2, S3).

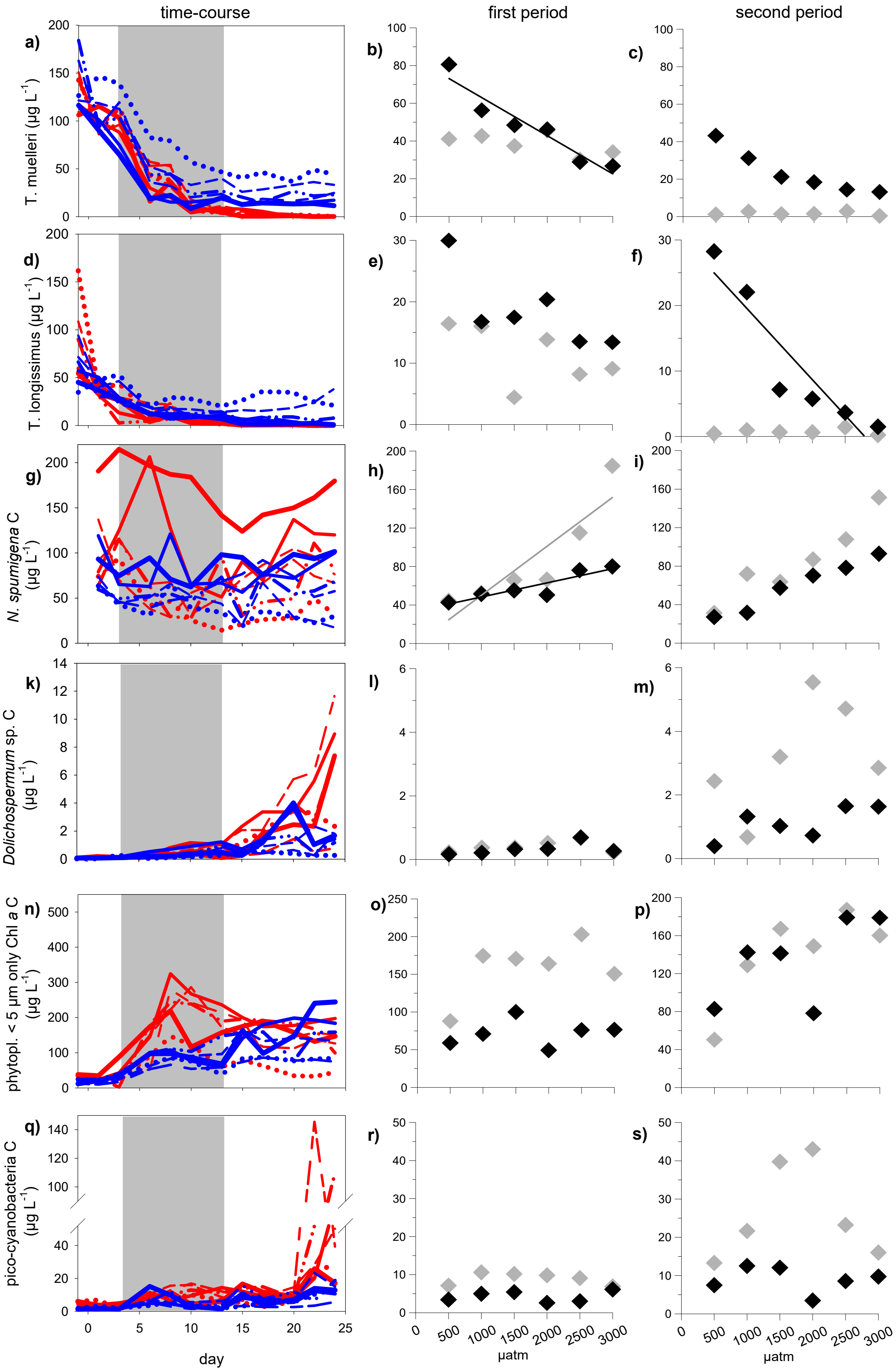
time-course
first period
second period
a)
b)
c)
d)
e)
f)
g)
h)
i)
k)
l)
m)
n)
o)
p)
q)
r)
s)
T. muelleri (µg L-1)
T. longissimus (µg L-1)
N. spumigena C (µg L-1)
Dolichospermum sp. C (µg L-1)
phytopl. < 5 µm only Chl a C (µg L-1)
pico-cyanobacteria C (µg L-1)
day
µatm

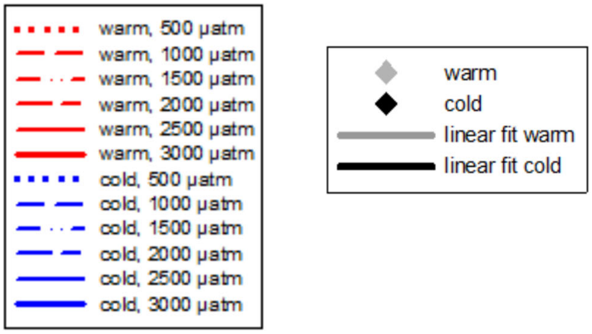

Figure S2. : Carbon (µg C L^{-1}) of inedible phytoplankton species/taxa, over the entire course of time and as mean values of the first and the second period. The first period, during which a bloom in the warm treatments occurred, is marked in grey color. a-c) *T.* muelleri, d-f) *T. longissimus*, g-i) *N. spumigena*, k-m) *Dolichospermum* sp., n-p) phytoplankton < 5µm only containing chl *a, q-s)* pico-cyanobacteria. Symbol attribution to treatment combinations (temperature treats + CO_2 target values) are given in the legends. Fitted lines indicate a significant response of phytoplankton relative contribution to CO_2 at the different temperature levels.

Supplement material*: Species / groups of the edible phytoplankton fraction:*

Both, carbon of the flagellates *P. cordatum*, *P. micans* and *Dinophysis* sp. was significantly higher in the cold treatments compared to the warm ones and additionally negatively responded to elevated CO_2 during the first period (Fig. S3 b, e, h; Table S2). During the second period, *P. cordatum* and *P. micans* were nearly disappeared in all warm and in the cold high CO_2 treatments (Fig. S3c, f; Table S2, S3). *Dinophysis* sp. carbon was also minor in all treatments in the second period, but decreased with elevated CO_2, with stronger response in the low temperature treatments (Fig. S2 i; Table S3, S4). In contrast to the other edible flagellates, *Teleaulax* sp. positively responded to elevated CO_2 during the first period (Fig. S2 l; Table S2). In the second period, carbon of *Teleaulax* sp. and the diatom *D. brightwellii* negatively responded to temperature, however differences between treatments were minor (Fig. S2 m, p; Table S3).

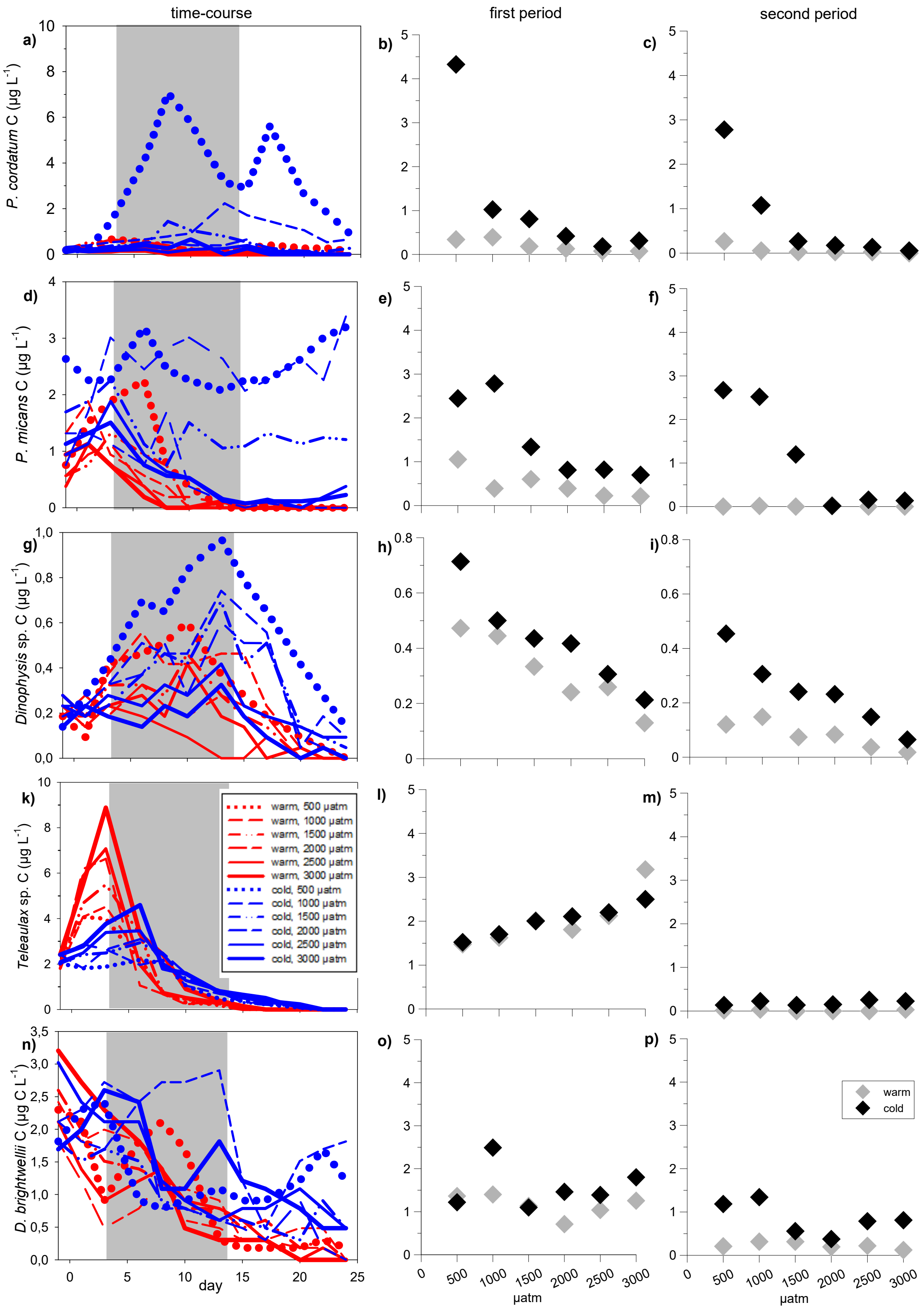

Figure S3. : Carbon (µg C L^{-1}) of edible phytoplankton species/taxa, over the entire course of time and as mean values of the first and the second period. The first period, during which a bloom in the warm treatments occurred, is marked in grey color. a-c) *P. cordatum*, d-f) *P. micans*, g-i) *Dinophysis* sp., k-m) *Teleaulax* sp., n-p) *D. brightwellii.* Symbol attribution to treatment combinations (temperature treats

+ CO_2 target values) are given in the legends. Fitted lines indicate a significant response of phytoplankton relative contribution to CO_2 at the different temperature levels.

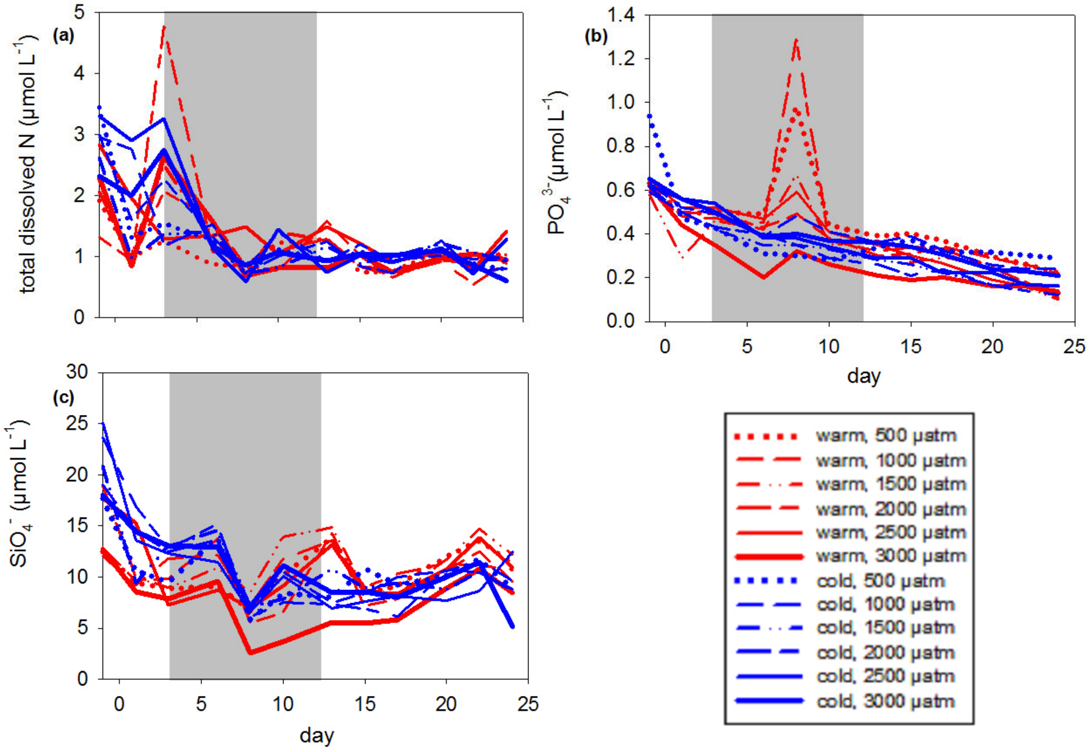

Figure S4. Time-course of dissolved inorganic nutrient concentrations (µmol L^{-1}) of: (a) total dissolved inorganic N (NO_2^- / NO_3^-, NH_4^+), (b) phosphate (PO_4^{3-}) and (c) silicate (SiO_4^-). Symbol attribution to treatment combinations (temperature treats + CO_2 target values) are given in the legend. The first period, during which a bloom in the warm treatments occurred, is marked in grey color.

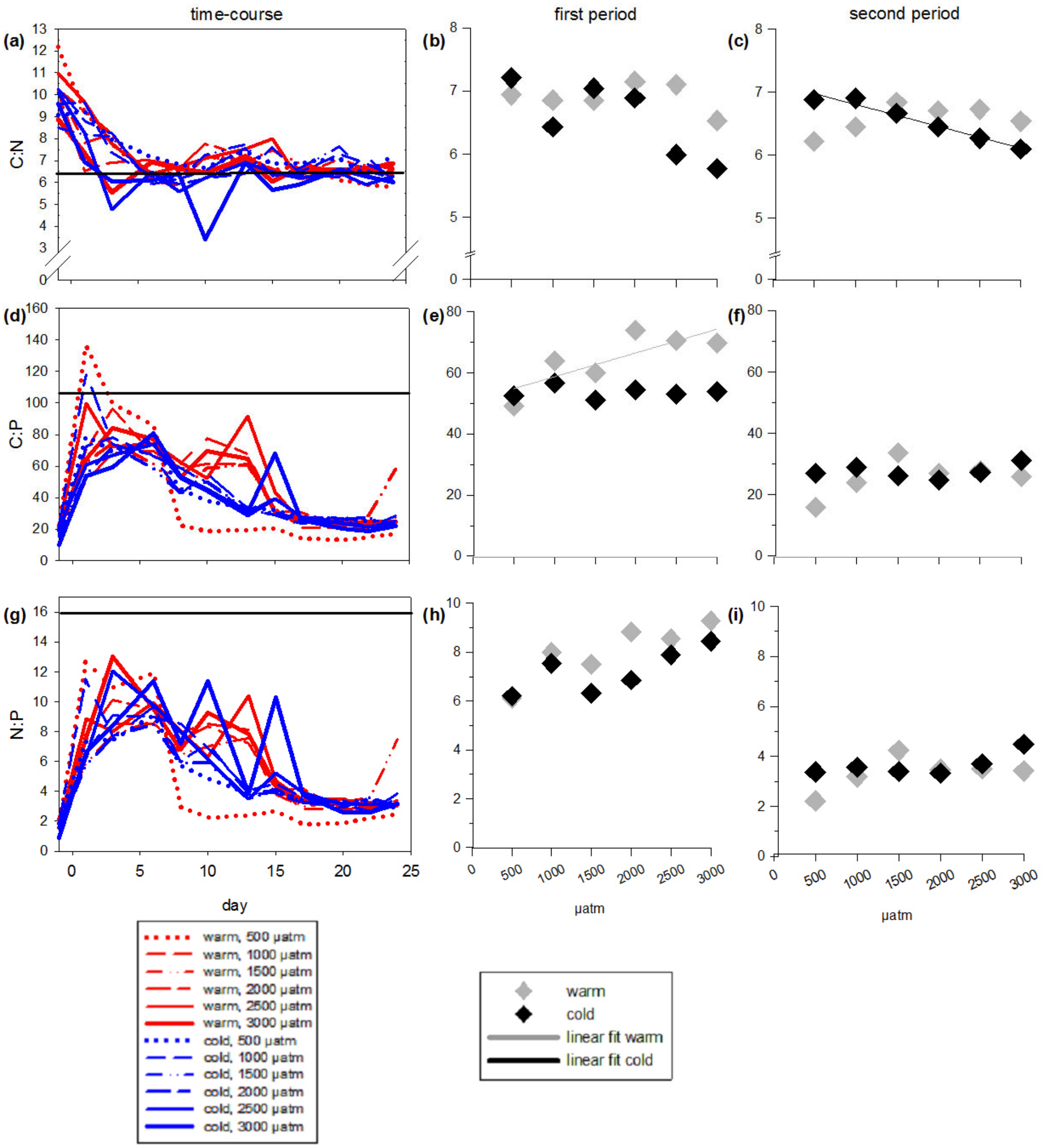

Figure S5. Plankton stoichiometry (mol:mol) over the entire course of time and as mean values of the first and the second period. The first period, during which a bloom in the warm treatments occurred, is marked in grey color. (a–c) carbon to nitrogen ratio (C:N), (d–f) carbon to phosphorus ratio (C:P), (g–i) nitrogen to phosphorus ratio (N:P). Symbol attribution to treatment combinations (temperature treats + CO_2 target values) are given in the legend. Redfield Ratios are marked with a horizontal line in the subpanels (a), (d) and (g). Fitted lines indicate a significant response of phytoplankton relative contribution to CO_2 at the different temperature levels.
