# Supplementary material for: Composition and Dominance of Edible and Inedible Phytoplankton Predict Responses of Baltic Sea Summer Communities to Elevated Temperature and CO2
Source: Microorganisms. 2021 Nov 4;9(11):2294. doi: 10.3390/microorganisms9112294 (PMC8621663; doi:10.3390/microorganisms9112294)
Supplement: Supplementary file 1 [file microorganisms-09-02294-s001.zip › microorganisms_1390211_Supplement Tables_statistical analyses_review_15.10.21.pdf]

Table S1: Results of generalized least squares model (gls) testing for the effect of temperature (T), CO_2, time and the interaction effect of temperature and CO_2 (T x CO_2), time and temperature (time x T), time and CO_2 (time x CO_2) and the interaction of time, temperature and CO_2 (time x T x CO_2) over the entire course of experimental time. Significant results are in bold. *$p \leq 0.05$, ** $p < 0.01$, *** $p < 0.001$.

Response variable	factor	df residual	t-value	*p*
(log) Total phytoplankton C	T	136	0.40650	0.6850
(µg L^{-1})	CO_2	136	-1.41829	0.1484
	time	136	-1.81855	0.0712
	T x CO_2	136	1.52885	0.1286
	time x T	136	-2.44663	**0.0157***
	time x CO_2	136	2.27327	**0.0246***
	time x T x CO_2	136	1.72325	0.0871
(log) inediblel phytoplankton C	T	136	0.46224	0.6446
(µg L^{-1})	CO_2	136	-1.39230	0.1661
	time	136	-1.84390	0.0674
	T x CO_2	136	1.51706	0.1316
	time x T	136	-2.23612	**0.0270***
	time x CO_2	136	2.44929	**0.0156***
	time x T x CO_2	136	1.55347	0.1226
Ediblel phytoplankton C	T	136	-1.722063	0.0873
(µg L^{-1})	CO_2	136	-1.406631	0.1618
	time	136	-0.113228	0.9100
	T x CO_2	136	1.019974	0.3096
	time x T	136	-4.374546	**<0.001*** **
	time x CO_2	136	-4.007652	**<0.001*** **
	time x T x CO_2	136	2.489965	**0.0140***
Inedible flagellates > 100 µm C	T	136	-0.136382	0.8917
(µg L^{-1})	CO_2	136	-2.221123	0.0280
	time	136	-4.241355	**<0.001*** **
	T x CO_2	136	0.105978	0.9158
	time x T	136	-2.153234	**0.0331***
	time x CO_2	136	-0.290570	0.7718
	time x T x CO_2	136	1.341232	0.1821
(log) filamentous cyanobacteria	T	136	-1.201710	0.2318
	CO_2	136	1.163015	0.2417
C (µg L^{-1})	time	136	-3.039261	**0.0029****
	T x CO_2	136	2.231858	**0.0274***
	time x T	136	1.463984	0.1457
	time x CO_2	136	2.758938	**0.0067****
	time x T x CO_2	136	-1.378534	0.1705
Phytoplankton < 5 µm	T	136	0.7633037	0.4466
C (µg L^{-1})	CO_2	136	-0.5613302	0.5755
	time	136	1. 3070283	0.1934
	T x CO_2	136	0.7633287	0.4466
	time x T	136	-0.0953977	0.9241
	time x CO_2	136	1.8449223	0.0672
	time x T x CO_2	136	-0.0862737	0.9314
Edible flagellates 5-100 µm C	T	136	-2.133546	**0.0347***
(µg L^{-1})	CO_2	136	-2.305907	**0.0226***
	time	136	-0.226604	0.8211
	T x CO_2	136	2.024677	**0.0449***
	time x T	136	-2.840604	**0.0052****
	time x CO_2	136	-2.860836	**0.0049****
	time x T x CO_2	136	1.523333	0.1300
Diatom C (µg L^{-1})	T	136	-0.111106	0.9117

	CO_2	136	1.240776	0.2168
	time	136	1.040369	0.3000
	T x CO_2	136	-1.576518	0.1172
	time x T	136	-4.124063	**<0.001*** **
	time x CO_2	136	-3.574167	**<0.001*** **
	time x T x CO_2	136	2.750142	**0.0068** **
T. muelleri C (μg L^{-1})	T	136	-1.876498	0.0627
	CO_2	136	-3.207634	**0.0017** **
	time	136	-5.623833	**<0.001*** **
	T x CO_2	136	1.778010	0.0776
	time x T	136	-0.608874	0.5436
	time x CO_2	136	0.709675	0.4791
	time x T x CO_2	136	-0.180114	0.8573
T. longissimus C (μg L^{-1})	T	136	2.418613	**0.0169***
	CO_2	136	-0.265923	0.7907
	time	136	-1.245801	0.2150
	T x CO_2	136	-2.377907	**0.0188***
	time x T	136	-3.947985	**<0.001*** **
	time x CO_2	136	-1.727249	0.0864
	time x T x CO_2	136	3.294387	**0.0013** **
(log) *N. spumigena* C (μg L^{-1})	T	101	2.505452	0.0138
	CO_2	101	0.975317	0.3317
	time	101	1.670423	0.0979
	T x CO_2	101	-2.127387	**0.0358***
	time x T	101	-7.362956	**<0.001*** **
	time x CO_2	101	-4.247658	**<0.001*** **
	time x T x CO_2	101	5.098848	**<0.001*** **
Dolichospermum sp. C (μg L^{-1})	T	136	0.651282	0.5160
	CO_2	136	1.984623	**0.0492***
	time	136	5.733659	**<0.001*** **
	T x CO_2	136	-0.873599	0.4346
	time x T	136	1.333287	0.1847
	time x CO_2	136	0.396260	0.6925
	time x T x CO_2	136	0.222790	0.8240
(log) Phytoplankton <5 μm only chl *a* C (μg L^{-1})	T	136	0.495375	0.6211
	CO_2	136	-0.592927	0.5542
	time	136	2.450446	**0.0155***
	T x CO_2	136	0.948159	0.3447
	time x T	136	-0.597510	0.5512
	time x CO_2	136	1.412443	0.1601
	time x T x CO_2	136	-0.118890	0.9055
(log) Pico-cyanobacteria C (μg L^{-1})	T	136	0.922033	0.3581
	CO_2	136	-0.704094	0.4826
	time	136	3.209547	**0.0017** **
	T x CO_2	136	0.827448	0.4094
	time x T	136	0.469603	0.6394
	time x CO_2	136	0.176067	0.8605
	time x T x CO_2	136	-0.153293	0.8784
(log) *P. cordatum* C (μg L^{-1})	T	100	1.902030	0.0600
	CO_2	100	3.244998	**0.0016** **
	time	100	2.651227	**0.0093** **
	T x CO_2	100	1.062248	0.2907
	time x T	100	-2.404264	**0.0180***
	time x CO_2	100	-2.004831	**0.0477***
	time x T x CO_2	100	1.032104	0.3045
(log) *P. micans*. C (μg L^{-1})	T	95	-0.531658	0.5962
	CO_2	95	-2.043635	**0.0438***
	time	95	2.8031170	**0.0061** **
	T x CO_2	95	-0.100642	0.9200
	time x T	95	-3.877390	**<0.001*** **
	time x CO_2	95	-5.531063	**<0.001*** **
	time x T x CO_2	95	1.710434	0.0904

(log)*Dinophysis* sp. C (μg L^{-1})	T	117	-0.5729424	0.5678
	CO_2	117	-1.6081819	0.1105
	time	117	-0.5294375	0.5975
	T x CO_2	117	0.1183348	0.9061
	time x T	117	-0.8114814	0.4187
	time x CO_2	117	-0.9800786	0.3291
	time x T x CO_2	117	0.5783711	0.5641
(log) *Teleaulax* sp. C (μg L^{-1})	T	97	0.592606	0.5548
	CO_2	97	0.995937	0.3218
	time	97	-5.818348	**<0.001*** **
	T x CO_2	97	-0.002460	0.9980
	time x T	97	-2.147911	**0.0342***
	time x CO_2	97	0.143754	0.8860
	time x T x CO_2	97	0.434634	0.6648
D. brightwellii C (μg L^{-1})	T	136	0.540498	0.5897
	CO_2	136	1.625296	0.1064
	time	136	-1.250117	0.2134
	T x CO_2	136	-1.009871	0.3144
	time x T	136	-2.357429	**0.0198***
	time x CO_2	136	-2.718498	**0.0074****
	time x T x CO_2	136	1.389876	0.1671
(log) C:N	T	136	0.10248	0.9185
	CO_2	136	-2.32444	**0.0216***
	time	136	-2.75305	**0.0067****
	T x CO_2	136	0.65283	0.5150
	time x T	136	-1.17532	0.2419
	time x CO_2	136	0.34342	0.7318
	time x T x CO_2	136	0.89226	0.3738
(log) C:P	T	135	0.073795	0.9413
	CO_2	135	-0.308615	0.7581
	time	135	-2.036997	**0.0436***
	T x CO_2	135	0.480963	0.6313
	time x T	135	-0.945909	0.3459
	time x CO_2	135	0.143486	0.8861
	time x T x CO_2	135	0.637329	0.5250
(log) N:P	T	135	0.025618	0.9796
	CO_2	135	0.275553	0.7833
	time	135	-1.176182	0.2416
	T x CO_2	135	0.274838	0.7839
	time x T	135	-0.517482	0.6057
	time x CO_2	135	0.004110	0.9649
	time x T x CO_2	135	0.322713	0.7474
Total dissolved N	T	136	-1.056225	0.2927
(μmol L^{-1})	CO_2	136	1.165196	0.2460
	time	136	-2.361684	**0.0196***
	T x CO_2	136	0.173842	0.8622
	time x T	136	0.862194	0.3901
	time x CO_2	136	-0.948315	0.3447
	time x T x CO_2	136	-0.090732	0.9278
PO_4^{3-}	T	136	2.785997	**0.0061****
(μmol L^{-1})	CO_2	136	0.046277	0.9632
	time	136	-1.127801	0.2613
	T x CO_2	136	-2.081611	**0.0393***
	time x T	136	-2.045220	**0.0428***
	time x CO_2	136	-0.217879	0.8279
	time x T x CO_2	136	1.495799	0.1370
Si_4^{2-}	T	136	-0.753690	0.4523
(μmol L^{-1})	CO_2	136	1.289303	0.1994
	time	136	-1.343862	0.1812
	T x CO_2	136	-1.132702	0.2593
	time x T	136	1.362630	0.1753
	time x CO_2	136	-1.504951	0.1347

time x T x CO_2	136	0.495248	0.6212

Table S2: Results of generalized least squares model (gls) testing for the effect of temperature (T), CO_2, and the interaction effect of temperature and CO_2 (T x CO_2) during the first period. Significant results are in bold. *p≤ 0.05, ** p< 0.01, *** p< 0.001.

Response variable	factor	df residual	t-value	*p*
% inedible flagellates > 100 µm	T	8	-3.938717	**0.0043****
on total phytopl. C	CO_2	8	-4.952595	**0.0011****
	T x CO_2	8	1.774507	0.1139
% edibleflagellates 5-100 µm	T	8	-5.186859	**<0.001*****
on total phytopl. C	CO_2	8	-5.028351	**0.0010****
	T x CO_2	8	2.562598	**0.0335***
% filamentous cyanobacteria	T	8	-0.845068	0.4226
on total phytopl. C	CO_2	8	3.644613	**0.0065****
	T x CO_2	8	0.766174	0.4656
% diatoms	T	8	-1.854671	0.1008
on total phytopl. C	CO_2	8	0.307481	0.7663
	T x CO_2	8	-1.364703	0.2095
% phytopl. < 5 µm	T	8	3.831281	**0.0050****
on total phytopl. C	CO_2	8	0.972288	0.3594
	T x CO_2	8	-1.773242	0.1141
(log) Total phytoplankton C	T	8	-0.73772	0.4818
(µg L^{-1})	CO_2	8	-0.86633	0.4115
	T x CO_2	8	3.73264	0.0058***
Inedible phytoplankton C	T	8	-0.808801	0.4420
(µg L^{-1})	CO_2	8	-0.635514	0.5428
	T x CO_2	8	4.519636	0.0020**
Edible phytoplankton C	T	8	-5.006703	0.0010**
(µg L^{-1})	CO_2	8	-3.675923	0.0063**
	T x CO_2	8	0.490532	0.6369
(log) Inedible flagellates >100 µm	T	8	-3.25951	**0.0115***
C (µg L^{-1})	CO_2	8	-5.12617	**<0.001****
	T x CO_2	8	2.35489	**0.0463***
(log) Filamentous cyanobacteria	T	8	-1.136689	0.2886
	CO_2	8	3.320493	**0.0105***
	T x CO_2	8	2.891799	**0.0201***
Phytoplankton < 5 µm C	T	8	1.4585787	0.1828
(µg L^{-1})	CO_2	8	0.2243038	0.8281
	T x CO_2	8	0.9180968	0.3854
(log) Edible flagellates 5-100 µm	T	8	-5.194249	0.008**
C (µg L^{-1})	CO_2	8	-4.684095	0.0016*
	T x CO_2	8	3.302352	0.0108*
Diatom C (µg L^{-1})	T	8	-1.992757	0.0834
	CO_2	8	-0.016442	0.9873
	T x CO_2	8	-0.375598	0.7170
T. muelleri C (µg L^{-1})	T	8	-5.004930	**<0.001*****
	CO_2	8	-7.271299	**<0.001*****
	T x CO_2	8	4.206430	**<0.001*****
T. longissimus C (µg L^{-1})	T	8	-1.920386	0.0911
	CO_2	8	-2.431088	**0.0411***
	T x CO_2	8	0.745374	0.4774
N. spumigena C (µg L^{-1})	T	8	-1.144287	0.2856
	CO_2	8	3.28363	**0.0111***
	T x CO_2	8	2.887418	**0.0203***

Dolichospermum sp. C ($\mu g\ L^{-1}$)	T	8	0.6752069	0.5186
	CO_2	8	1.3080453	0.2272
	T x CO_2	8	-0.4429917	0.6695
Phytoplankton < 5 µm only chl *a* C ($\mu g\ L^{-1}$)	T	8	1.3756529	0.2062
	CO_2	8	0.2156383	0.8347
	T x CO_2	8	0.9926941	0.3499
Pico-cyanobacteria C ($\mu g\ L^{-1}$)	T	8	2.6358914	**0.0299***
	CO_2	8	0.3342436	0.7468
	T x CO_2	8	-0.5469482	0.5993
(log) *P. cordatum* C ($\mu g\ L^{-1}$)	T	8	-3.897752	**0.0046****
	CO_2	8	-5.693473	**<0.001*****
	T x CO_2	8	1.365857	0.2091
(log) *P. micans* C ($\mu g\ L^{-1}$)	T	8	-3.188228	**0.0128***
	CO_2	8	-4.480483	**0.0021****
	T x CO_2	8	0.089412	0.9310
Dinophysis sp.C ($\mu g\ L^{-1}$)	T	8	-3.307566	**0.0107***
	CO_2	8	-8.436744	**<0.001*****
	T x CO_2	8	1.424640	0.1921
Teleaulax sp.C ($\mu g\ L^{-1}$)	T	8	-0.908663	0.3901
	CO_2	8	3.122997	**0.0142***
	T x CO_2	8	1.122079	0.2944
D. brightwellii C ($\mu g\ L^{-1}$)	T	8	0.385084	0.7102
	CO_2	8	0.009844	0.9924
	T x CO_2	8	-0.387490	0.7085
(log) C:N	T	8	-0.956588	0.3668
	CO_2	8	-3.127289	**0.0141***
	T x CO_2	8	1.955480	0.0863
C:P	T	8	-0.447313	0.6665
	CO_2	8	0.020187	0.9844
	T x CO_2	8	2.586698	**0.0323***
N:P	T	8	0.287896	0.7807
	CO_2	8	2.491033	**0.0375***
	T x CO_2	8	0.827418	0.4320

Table S3: Results of generalized least squares model (gls) testing for the effect of temperature (T), CO_2 and the interaction effect of temperature and CO_2 (T x CO_2) during the second period. Significant results are in bold. *$p \leq 0.05$, ** $p < 0.01$, *** $p < 0.001$.

Response variable	factor	df residual	t-value	*p*
Total phytoplankton C ($\mu g\ L^{-1}$)	T	8	-1.423629	0.1924
	CO_2	8	1.588208	0.1509
	T x CO_2	8	1.870230	0.0984
Inedible phytoplankton C ($\mu g\ L^{-1}$)	T	8	-1.269216	0.2400
	CO_2	8	1.774299	0.1139
	T x CO_2	8	1.791950	0.1109
(log) Edible phytoplankton C ($\mu g\ L^{-1}$)	T	8	-5.006703	**0.0010****
	CO_2	8	-3.675923	**0.0063****
	T x CO_2	8	0.490532	0.6369
(log) Inedible flagellates > 100 µm C ($\mu g\ L^{-1}$)	T	8	-5.097679	0.009**
	CO_2	8	-2.672748	0.0282*
	T x CO_2	8	1.220660	0.2570
(log) Filamentous cyanobacteria C ($\mu g\ L^{-1}$)	T	8	1.599105	0.1485
	CO_2	8	6.531437	**<0.001*****
	T x CO_2	8	0.119481	0.9087
Phytoplankton < 5 µm C ($\mu g\ L^{-1}$)	T	8	0.052991	0.9590
	CO_2	8	1.450211	0.1850

	T x CO_2	8	0.412471	0.6908
(log) Edible flagellates 5-100 µm C (µg L^{-1})	T	8	-6.693784	**<0.001*****
	CO_2	8	-6.924401	**<0.001*****
	T x CO_2	8	1.273975	0.2384
(log) Diatom C (µg L^{-1})	T	8	-3.154914	**0.0135***
	CO_2	8	-1.531894	0.1641
	T x CO_2	8	-0.244293	0.8132
(log) *T. muelleri* C (µg L^{-1})	T	8	-4.420674	**0.0022****
	CO_2	8	-1.918598	0.0913
	T x CO_2	8	0.524755	0.6140
(log) *T. longissimus* C (µg L^{-1})	T	8	-6.391495	**<0.001*****
	CO_2	8	-4.789403	**0.0014****
	T x CO_2	8	3.052888	**0.0158***
(log) *N. spumigena* C (µg L^{-1})	T	8	0.061508	0.9525
	CO_2	8	5.413742	**<0.001*****
	T x CO_2	8	1.991594	0.0816
Dolichospermum sp. C (µg L^{-1})	T	8	0.725673	0.4888
	CO_2	8	0.6850857	0.5126
	T x CO_2	8	0.6783824	0.5167
Phytoplankton < 5 µm only chl *a* C (µg L^{-1})	T	8	-0.2342063	0.8207
	CO_2	8	1.7470336	0.1188
	T x CO_2	8	0.4161874	0.6882
(log) Pico-cyanobacteria C (µg L^{-1})	T	8	1.200001	0.2645
	CO_2	8	-0.245088	0.8126
	T x CO_2	8	0.370596	0.7206
(log) *P. cordatum* C (µg L^{-1})	T	7	-4.251501	**0.0038****
	CO_2	7	-6.426122	**<0.001*****
	T x CO_2	7	1.171489	0.2797
(log) *P. micans* C (µg L^{-1})	T	4	-1.7314318	0.1584
	CO_2	4	-2.0778204	0.1963
	T x CO_2	4	0.9579617	0.3923
Dinophysis sp.C (µg L^{-1})	T	8	-8.143823	**<0.001*****
	CO_2	8	-9.722616	**<0.001*****
	T x CO_2	8	4.513311	**0.0020****
(log) *Teleaulax* sp. C (µg L^{-1})	T	5	-4.854862	**0.0047****
	CO_2	5	1.182049	0.2903
	T x CO_2	5	0.111846	0.9153
(log) *D. brightwellii* C (µg L^{-1})	T	8	-2.3473104	**0.0469***
	CO_2	8	-1.1657632	0.2773
	T x CO_2	8	-0.0719142	0.9444
C:N	T	8	-3.84145	**0.0049****
	CO_2	8	-4.58708	**0.0018****
	T x CO_2	8	4.47006	**0.0021****
C:P	T	8	-1.058106	0.3209
	CO_2	8	0.388403	0.7078
	T x CO_2	8	0.820492	0.4357
N:P	T	8	-0.510356	0.6236
	CO_2	8	1.394792	0.2006
	T x CO_2	8	0.056534	0.9563

Table S4: Results of generalized least sqares model (gls) testing for the effect of CO_2 under high and low temperature for the edible and inedible species separately. Significant results are in bold. *$p \leq 0.05$, ** $p < 0.01$, *** $p < 0.001$.

Response variable	factor	df residual	t-value	*p*
T. muelleri C first period (μg L^{-1})	CO_2 warm	4	-1.496190	0.2089
	CO_2 cold	4	-6.5866618	**0.0028****
T. longissimus C second period (μg L^{-1})	CO_2 warm	4	0.0881402	0.9340
	CO_2 cold	4	-4.863399	**0.0083****
N. spumigena C bloom (μg L^{-1})	CO_2 warm	4	6.141194	**0.0036****
	CO_2 cold	4	4.38497	**0.0118***
P. micans C second period (μg L^{-1})	CO_2 warm	4	0.6741999	0.5371
	CO_2 cold	4	-0.6298045	0.5630
P. cordatum C second period (μg L^{-1})	CO_2 warm	4	-0.3194113	0.7654
	CO_2 cold	4	-0.1399214	0.8955
Dinophysis sp. C second period (μg L^{-1})	CO_2 warm	4	-4.426824	**0.0115***
	CO_2 cold	4	-8.128184	**0.0012****
